# Patterns of antibiotic use for acute respiratory infections in under-three-year-old children in India: A cross-sectional study

**DOI:** 10.7189/jogh.13.04159

**Published:** 2023-12-22

**Authors:** Hector Gonzalez Dorta, Arijit Nandi

**Affiliations:** Institute for Health and Social Policy & Department of Epidemiology, Biostatistics, and Occupational Health, McGill University, 2001 McGill College Ave, Montreal, Quebec, Canada

## Abstract

**Background:**

Despite its mostly viral etiology, antibiotics are frequently used to treat acute respiratory infections (ARIs) in children. India is one of the largest global consumers of antibiotics and has one of the highest rates of resistance to antimicrobial treatments. However, the epidemiology of antibiotic treatment among young children in India is poorly understood.

**Methods:**

Using nationally representative household survey data from the Indian National Family Health Surveys (NFHS) conducted between 2015 and 2016 and 2019 and 2021, we estimated the prevalence of antibiotic use among 17 472 children under the age of three who reported ARI symptoms within two weeks before their mothers were interviewed. To assess the factors associated with antibiotic use for the treatment of ARI symptoms, we used multivariable logistic regression models that included sociodemographic, child-related, household, and health care related characteristics, with results reported on the prevalence difference (PD) scale.

**Results:**

We estimated that 18.7% (95% CI = 17.8-19.6) of under-three-year-old (U3) children who exhibited ARI symptoms in the two weeks prior to the survey were given antibiotics as a treatment. The highest prevalence was observed in the southern and northern geographic zones of India. Furthermore, multivariable regression models indicated that children with greater access to health services were more likely to receive antibiotics for ARI treatment, regardless of the type of health care facility (public, private or pharmacy/unregulated). Additionally, the prevalence of antibiotic consumption was higher among children from families with religious affiliations other than Muslim and Hindu backgrounds (i.e. Christian, Sikh, Buddhist/neo-Buddhist, Jain, Jewish, Parsi, no religion and other) (PD = 11.7 (95% CI = 6.3-16.7)) compared to Hindu families and among mothers with a secondary or higher education (PD = 5.8 (95% CI = 1.7-9.9)) compared to mothers lacking formal education.

**Conclusions:**

Our findings provide an important baseline for monitoring the use of antibiotics for the treatment of acute respiratory infections, and for designing interventions to mitigate potential misuse among young children in India.

Acute respiratory infections (ARIs) are a heterogeneous group of diseases caused by different pathogens that primarily affect the respiratory system. ARIs are categorised into upper respiratory tract infections (URTIs) and lower respiratory tract infections (LRTIs) depending on the site of infection [1]. ARIs are among the leading causes of childhood mortality worldwide, especially in low- and middle-income countries (LMICs). In 2019, an estimated 740 000 deaths among under-five-year-old (U5) children were attributed to LRTIs, making lower respiratory infections the leading cause of mortality among children aged 1-59 months [2]. Sub-Saharan Africa and South Asia are most affected by ARIs [3]. In India, there has been only a 1.6% reduction in the percentage of total deaths attributed to LRTIs between 2010 and 2019 [4].

Diagnosis of ARIs is often based on recognition of symptoms, which is difficult given the similar clinical presentation associated with distinct etiological agents [5]. Nucleic acid amplification techniques, mainly polymerase chain reaction (PCR) and its derivatives, are starting to be considered the new “gold standard” for confirmation, compared to cell culture techniques however, their use in poor resources settings is limited [6,7].

The treatment for children presenting with symptoms of ARI is usually symptomatic and supportive. The Indian and international guidelines give strong evidence for supportive care including oxygen and intravenous fluids when needed [8-10]. However, the routine use of antibiotics in children presenting with symptoms of ARIs is not recommended, unless there is evidence of bacterial infection [8-11]. This is because the majority of ARIs in children are caused by viruses, and coinfections with bacteria are rare [6,12]. Despite this, ARIs are the largest reason for antibiotic consumption in U5 children in LMICs [13]. The unnecessary use of antibiotics is associated with an increase in the cost of treatment, adverse reactions, and the development of bacterial antimicrobial resistance (AMR) in the community and geographic region [14].

AMR has been recognised as a major global health threat by the World Health Organization (WHO), with an estimated 1.27 million deaths directly attributable to bacterial AMR in 2019 [14,15]. It is projected that AMR will account for 10 million deaths per year by 2050 [14]. India is the world’s most populated country and is known to be the highest consumer of antibiotics in the world [16], with worryingly high levels of AMR [17].

Political commitment and efforts to contain AMR in India started with the health ministers of the South-East Asia Region’s Member States signing the Jaipur declaration in 2011, giving priority to AMR containment in national policy-making [18]. In April 2017, the Union Ministry of Health and Family Welfare launched India’s National Action Plans on AMR (NAP-AMR) which encompasses the different One Health strategic priorities to tackle AMR [19]. Nevertheless, implementation of AMR mitigation strategies has varied across the country and progress has been hampered by poor enforcement, inadequate multisectoral coordination, and the lack of financial allocation across states and union territories, which are responsible for health administration in India [20,21].

Several efforts have been made to restrict access to over-the-counter (OTC) antibiotics, such as the Schedule H1 policy ratified in 2013, before the NAP-AMR [22]. This policy aimed to limit the OTC sales of certain drugs to prescription-only drugs, including third- and fourth-generation cephalosporins, carbapenems, newer fluoroquinolones, and first- and second-line drugs for tuberculosis. The Schedule H1 also included the adequate labeling of these drugs and a separate register of the prescription-based sales. Despite the significant decrease in the overall retail sales of antimicrobial drugs [23], research indicates that implementation varied across states and did not reduce OTC antibiotic use [24].

In 2013 the Indian Council of Medical Research (ICMR) founded the Antimicrobial Resistance Surveillance & Research Network (AMRSN) across selected hospitals in India [25]. Despite the great effort made and the positive implementation results, the ICMR-AMRSN does not provide information on antibiotic consumption and its coverage is limited to tertiary care hospitals, which does not represent the situation and practices in local settings. Therefore, there is a need to characterise patterns of antibiotic use at the community level in India [25].

In this study, we employed nationally representative household survey data from the National Family Health Survey (NFHS) to: 1) describe the national and regional patterns of antibiotic use for the treatment of ARI in under-three-year-old (U3) children; 2) examine ARI treatment seeking-behaviour and attitudes in urban and rural environments; and 3) identify predictors of antibiotic use for ARI treatment in U3 Indian children using multivariable regression models. As such, this study aims to improve understanding of antibiotic consumption patterns in India and provide insights for addressing the challenges posed by antimicrobial resistance.

## METHODS

### Sample

In this serial cross-sectional study, we used data from 2015-2016 (NFHS-4) and 2019-2021 (NFHS-5) NFHS surveys. The NFHS surveys are developed under the Demographic and Health Survey (DHS) program which is responsible for collecting, analysing, and disseminating accurate and representative data on population health in more than 90 LMICs using funds from the United States Agency for International Development (USAID), UNICEF, WHO and other international organisations [26].

The NFHS is a cross-sectional household demographic and health survey conducted under the stewardship of the Ministry of Health and Family Welfare and coordinated by the International Institute of Population Sciences (IIPS), Mumbai. Conducted regularly since 1991, the NFHS is a household survey that covers all states and union territories and is conducted using a two-stage stratified sampling scheme. It is designed to provide reliable information on population health and nutritional status at the district, state, and national levels [27,28].

The NFHS-4 was conducted from 20 January 2015 to 4 December 2016 and included information from 601 509 households. In these households, 699 686 ever-married women aged 15-49 were interviewed, with a response rate of 97 percent [27]. The NFHS-5 fieldwork was conducted in two phases, phase one from 17 June 2019 to 30 January 2020 and phase two from 2 January 2020 to 30 April 2021. In the 636 399 households surveyed, 724 115 ever-married women aged 15-49 were interviewed, with a response rate of 97 percent [28]. Further details on the sampling design and survey procedures are provided in the NFHS-4 and NFHS-5 national reports [27,28]. Moreover, these questionnaires are constantly revised to include the latest standards and global recommendations [29]. Likewise, a WHO team validated the wide use of DHS surveys and their impact in public health policies in LMIC [30] and several studies have been published using NFHS data in the context of acute respiratory infections [31-34].

### Sample selection

**Figure 1:**
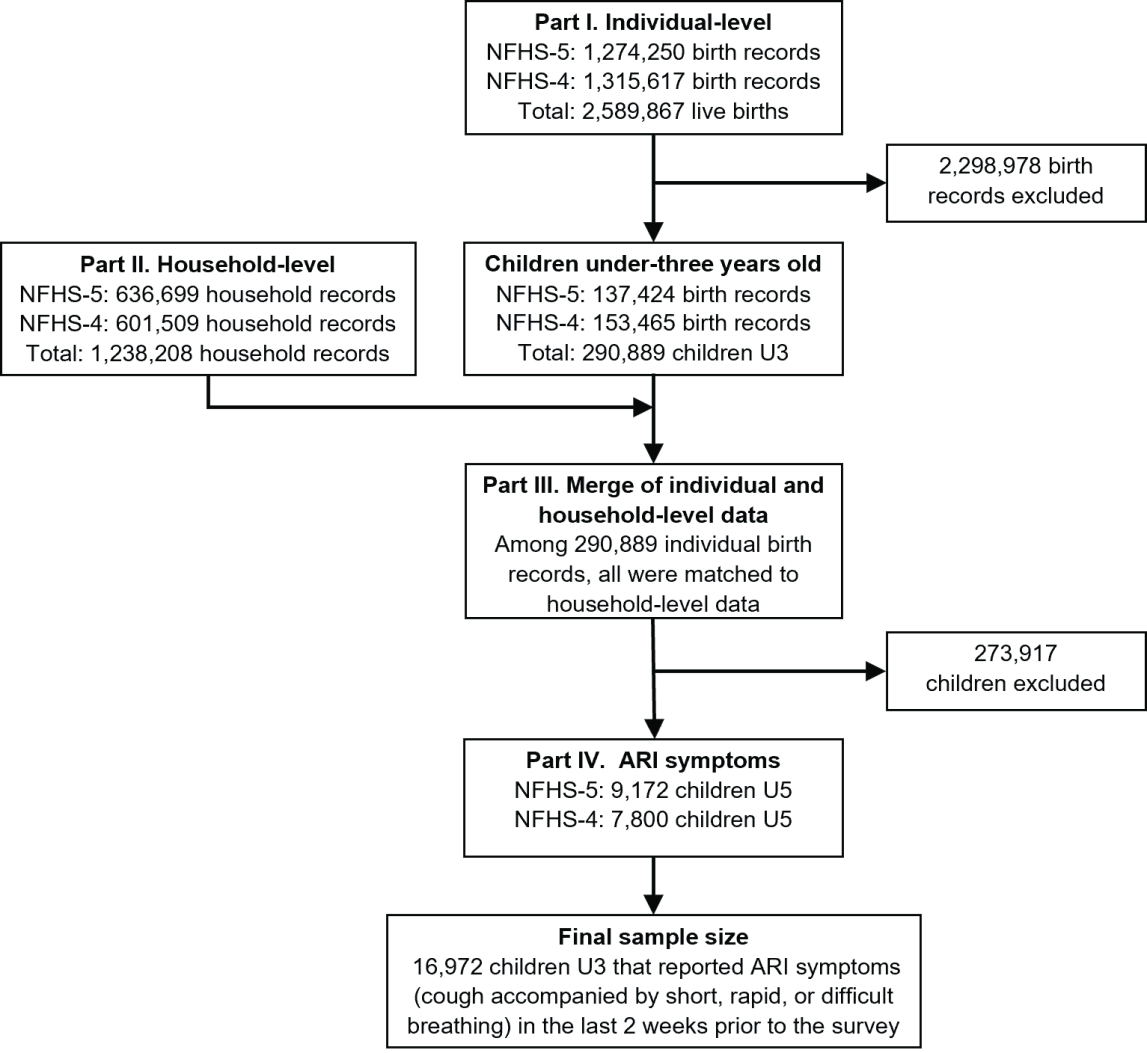
Sample selection. The final unweighted sample size (n = 16 972) was obtained through the combination of individual birth records and household information from the NFHS-4 and NFHS-5 women’s and household questionnaires.

We merged individual-level and household-level information from the women’s and household questionnaires, respectively, as shown in **Figure 1**. Questions on child immunisations and health status from the women’s questionnaire were asked of all live births, surviving to the time of the survey, in the previous five years in NFHS-4 and three years in NFHS-5. To pool data across both waves, we restricted our sample to children under three years (36 months) of age, resulting in a final sample size of 16 972 children under three years of age who reported ARI symptoms in the last two weeks.

### Measures

#### Outcome variable

Our primary outcome variable was the proportion of under-three children with ARI symptoms in the previous two weeks before the survey was conducted who were treated with antibiotics. The NFHS does not record data from physicians. U3 children with ARI symptoms were identified using two questions from the women’s questionnaire: 1) “has (NAME) had an illness with a cough at any time in the last 2 weeks?”; (2) “when (NAME) had an illness with a cough, did (he/she) breathe faster than usual with short, rapid breaths or have difficulty breathing?”. Consistent with prior research [3,8,32,35], ARI was defined as cough accompanied by short, rapid, or difficult breathing reported in the two weeks prior to the survey. If mothers reported that their child had cough symptoms, they were asked about the treatment received, specifically “what drugs did (NAME) take? Any other drugs?”. A detailed list of antimalarial drugs, antibiotic drugs (pills, syrups, or injections), and other treatments was given. A dichotomous variable measuring antibiotic consumption was created, coded as “1” if the child took antibiotic drugs or “0” if the child did not take antibiotic drugs for the treatment of the ARI symptoms.

#### Predictor variables

To identify characteristics associated with antibiotic use, we included a list of potential predictor variables based on the existing literature and expert opinion [32,36-39]. The predictor variables were classified into four categories: sociodemographic variables, child-related variables, household variables, and health care variables. The definition for each variable can be found in the Table S1 in the **Online Supplementary Document**.

### Statistical analyses

Our inferential goals were to describe the characteristics of children with reported symptoms of ARI in the past two weeks, the treatment they received, and the prevalence and predictors of receiving antibiotics for treatment. We used standard descriptive statistics to describe our study sample, reporting means and standard errors for continuous variables and frequencies and percentages for categorical variables. Given important rural vs urban differences in child health in India [40], descriptive analyses of potential predictors were stratified by whether the household was in a rural vs urban area.

The prevalence of antibiotic treatment was defined as the proportion of U3 children with ARI symptoms in the two weeks prior to the interview who were given antibiotics for treatment. We mapped the prevalence of receiving antibiotics for treatment across administrative zones in India using QGIS 3.16.4-Hannover [41]. The map was created using the geospatial data for the NFHS-4 survey which was downloaded from the DHS Program Spatial Data Repository [26]. To account for recent changes in the regional organisation of Indian states and union territories and to pool and compare results from NFHS-4 and NFHS-5, the union territory of Ladakh was considered as part of the Jammu and Kashmir state. Moreover, the union territories of Dadra and Nagar Haveli and Daman and Diu boundaries and corresponding information in NFHS-4 were merged into a single region [42,43].

We used multivariable logistic regression models to estimate the adjusted associations between our predictors of interest and the probability of being given antibiotics for treatment among U3 children with ARI symptoms. We ran four sequential models, starting from the most “upstream” or distal predictors and subsequently adding groups of variables that were more proximal to the experience of ARI and measurement of the outcome, with the intention of obtaining a final, fully adjusted logistic regression model.

In the first model, we included only sociodemographic characteristics, followed by household variables in the second model, child-related predictors in the third model, and finally health care-related characteristics in the fourth model. To facilitate interpretation of our results, we measured associations between our predictors and outcome on the absolute scale by calculating prevalence differences with their corresponding 95% confidence intervals (CIs) [44,45]. The prevalence difference estimates were obtained from the calculation of the average marginal effect of each predictor using the R packages “survey” and “margins” [46,47]. Entries containing any missing values were excluded from the models.

All statistics, including measures of frequency and association, accounted for the clustering of observations within PSUs and the complex sampling design of the NFHS (i.e. stratification and clustering). All results were weighted to account for the under- and oversampling of certain regions to produce population-representative estimates with robust standard errors. In strata defined by state and urban/rural with a single PSU, the “lonely” PSU was treated as “certain” and did not contribute to the variance computation of the strata. Statistical analyses were executed in RStudio version 4.1.2 [48] and the statistical code is available via the GitHub repository.

## RESULTS

### Descriptive analysis

Our final weighted sample size included 8958 (51.3%) birth records from the 2015-2016 NFHS-4 and 8494 (48.7%) birth records from the 2019-2021 NFHS-5, resulting in an analytic sample of 17 452 U3 children with reported ARI symptoms in the two weeks prior to the survey interview. The weighted distributions of the sociodemographic and household characteristics of the children and their mothers are shown in **Table 1**. Briefly, the mean age of the children in our sample was 17 months and 9600 (55%) were male. Respondents from rural areas were, on average, more socioeconomically disadvantaged, as reflected by the wealth index and distribution of household variables. Nearly one in five, or 18.7% (95% CI = 17.8-19.6), of children with ARI symptoms were given antibiotics as a treatment, with a slightly higher prevalence in urban (21.3%) compared to rural (17.9%) areas. The prevalence of receiving antibiotics was 20.2% (95% CI = 18.8-21.5) in the NFHS-4 and 17.2% (95% CI = 15.0-18.5) in the NFHS-5. The unweighted sample characteristics are shown in Table S2 in the **Online Supplementary Document**.

**Table 1:** Distribution of sociodemographic characteristics, household variables, child-related variables, and health care variables of under-three-year-old children with symptoms of ARI in the two weeks preceding the survey interview*

	Overall†	Urban†	Rural†
	n = 17 452	n = 4047 (23.2%)	n = 13 405 (76.8%)
**Outcome variable**			
**Were given antibiotics**	3266 (18.7%)	860 (21.3%)	2405 (17.9%)
**Sociodemographic variables**			
**Wealth index (quintiles)**			
Q1 (poorest)	4918 (28.2%)	206 (5.1%)	4712 (35.2%)
Q2	4156 (23.8%)	430 (10.6%)	3726 (27.8%)
Q3	3385 (19.4%)	858 (21.2%)	2527 (18.8%)
Q4	2899 (16.6%)	1274 (31.5%)	1625 (12.1%)
Q5 (wealthiest)	2093 (12.0%)	1278 (31.6%)	815 (6.1%)
**Religion**			
Hindu	13 960 (80.0%)	3004 (74.2%)	10 956 (81.7%)
Muslim	2826 (16.2%)	880 (21.8%)	1946 (14.5%)
Others	666 (3.8%)	162 (4.0%)	503 (3.8%)
**Caste/tribe**			
Scheduled caste	4152 (23.8%)	866 (21.4%)	3286 (24.5%)
Schedule tribe	1627 (9.3%)	152 (3.7%)	1475 (11.0%)
Other backward classes	7862 (45.0%)	1903 (47.0%)	5959 (44.5%)
None	3074 (17.6%)	953 (23.5%)	2121 (15.8%)
Missing	738 (4.2%)	174 (4.3%)	564 (4.2%)
**Mother’s age (years)**	25.7 (±4.7)	26.1 (±4.5)	25.5 (±4.7)
**Mother’s educational level**			
No education	4143 (23.7%)	502 (12.4%)	3641 (27.2%)
At least some primary	2486 (14.2%)	478 (11.8%)	2009 (15.0%)
Incomplete secondary	7824 (44.8%)	1869 (46.2%)	5955 (44.4%)
Secondary or higher	2999 (17.2%)	1198 (29.6%)	1801 (13.4%)
**Household variables**			
**Household size**	6.5 (±2.9)	6.3 (±2.9)	6.4 (±2.9)
**Any previous children deceased**	1728 (9.9%)	252 (6.2%)	1476 (11.0%)
**Type of cooking fuel**			
Clean	6125 (35.1%)	2908 (71.9%)	3216 (24.0%)
Unclean	10 154 (58.2%)	880 (21.7%)	9275 (69.2%)
Missing	1173 (6.7%)	259 (6.4%)	914 (6.8%)
**Source of drinking water**			
Improved	15 469 (88.6%)	3707 (91.6%)	11 762 (87.7%)
Unimproved	776 (4.4%)	70 (1.7%)	706 (5.3%)
Missing	1207 (6.9%)	270 (6.7%)	937 (7.0%)
**Toilet facility**			
Improved	9357 (53.6%)	3106 (76.8%)	6250 (46.6%)
Unimproved	6883 (39.4%)	654 (16.2%)	6228 (46.5%)
Missing	1213 (6.9%)	287 (7.1%)	926 (6.9%)
**Soap for handwashing**			
Yes	10 775 (61.7%)	3207 (79.2%)	7568 (56.5%)
Missing	361 (2.1%)	58 (1.4%)	303 (2.3%)
**Media accessibility**			
Not at all	4923 (28.2%)	403 (10.0%)	4521 (33.7%)
Less than once a week	2929 (16.8%)	532 (13.1%)	2397 (17.9%)
At least once a week	5027 (28.8%)	1480 (36.6%)	3547 (26.5%)
Almost every day	4572 (26.2%)	1632 (40.3%)	2940 (21.9%)
**Below poverty line card**			
Yes	7306 (41.9%)	1189 (29.4%)	6116 (45.6%)
Missing	34 (0.2%)	8 (0.2%)	26 (0.2%)
**Smoke exposure**			
Never	8931 (51.2%)	2393 (59.1%)	6538 (48.8%)
Daily	5474 (31.4%)	1067 (26.4%)	4408 (32.9%)
Weekly or less	3047 (17.5%)	588 (14.5%)	2459 (18.3%)
**Child-related variables**			
**Sex**			
Male	9605 (55.0%)	2236 (55.2%)	7369 (55.0%)
Female	7847 (45.0%)	1811 (44.8%)	6036 (45.0%)
**Age (months)**	17.0 (±9.7)	17.4 (±9.7)	16.9 (±9.7)
**Birth order**	2.2 (±1.4)	1.9 (±1.2)	2.2 (±1.4)
**Place of delivery**			
Public	10 421 (59.7%)	2091 (51.7%)	8330 (62.1%)
Private	4326 (24.8%)	1585 (39.2%)	2741 (20.4%)
Home	2594 (14.9%)	332 (8.2%)	2262 (16.9%)
Other/NGO	111 (0.6%)	39 (1.0%)	71 (0.5%)
**Stunting (height/age)**			
Not stunted	10 291 (59.0%)	2589 (64.0%)	7702 (57.5%)
Moderately stunted	3144 (18.0%)	594 (14.7%)	2549 (19.0%)
Severely stunted	2666 (15.3%)	487 (12.0%)	2179 (16.3%)
Missing	1351 (7.7%)	377 (9.3%)	974 (7.3%)
**Wasting (weight/height)**			
Not wasted	12 477 (71.5%)	2890 (71.4%)	9588 (71.5%)
Moderately wasted	2218 (12.7%)	426 (10.5%)	1792 (13.4%)
Severely wasted	1323 (7.6%)	327 (8.1%)	996 (7.4%)
Missing	1434 (8.2%)	405 (10.0%)	1028 (7.7%)
**Healthcare variables**			
**Problems accessing health care**	5.2 (±2.6)	4.2 (±2.9)	5.4 (±2.5)
**Health worker visit in last 3 months**	10 667 (61.1%)	2105 (52.0%)	8562 (63.9%)
**Covered by health insurance**	3157 (18.1%)	694 (17.1%)	2463 (18.4%)
**Has vaccination card**			
No card	1792 (10.3%)	357 (8.8%)	1435 (10.7%)
Yes, seen	13 132 (75.2%)	3094 (76.4%)	10 039 (74.9%)
Yes, not seen	2528 (14.5%)	596 (14.7%)	1931 (14.4%)
**Fully immunised**			
Yes	8229 (47.2%)	1945 (48.1%)	6284 (46.9%)
Missing	29 (0.2%)	3 (0.1%)	26 (0.2%)
**Anganwadi or ICDS benefits**	12 296 (70.5%)	2319 (57.3%)	9976 (74.4%)
**Drugs for intestinal parasites (last 6 months)**			
Yes	5861 (33.6%)	1338 (33.1%)	4523 (33.7%)
Missing	95 (0.5%)	12 (0.3%)	83 (0.6%)
**Vitamin A supplementation (last 6 months)**			
Yes	9963 (57.1%)	2304 (56.9%)	7659 (57.1%)
Missing	157 (0.9%)	32 (0.8%)	126 (0.9%)
**Iron supplementation (last 7 days)**			
Yes	5481 (31.4%)	1272 (31.4%)	4209 (31.4%)
Missing	80 (0.5%)	10 (0.2%)	70 (0.5%)

ARI – acute respiratory infection, NGO – non-governmental organisation, ICDS – integrated child development services

*NFHS-4 and NFHS-5; n = 17 452.

†Mean (±standard deviation (SD)) or frequency (%).

### Treatment of ARI

We assessed treatment-seeking behaviours reported by mothers of children with symptoms of ARI (**Table 2**). Nearly half (46.5%) of respondents first sought advice or treatment for their child in a private health facility. We observed differences between the urban and rural samples, with 50.2 and 45.4%, respectively, seeking treatment in private health facilities (Pearson χ^2^ *P* < 0.001). Moreover, the use of pharmacy or unregulated, traditional methods was more common within the rural sample (7.4%) compared to the urban (4.7%) sample. Regarding the start of the treatment, children in urban environments tended to receive treatment slightly earlier, 1.0 (±1.0) (standard deviation (SD) = 1.0) days after the onset of symptoms, than children in rural environments, where the mean was 1.3 (±1.3). We did not observe differences concerning the amount of food or drink given to the children while ill. Overall, one-half of the children received less food or drink. The corresponding unweighted statistics are shown in Table S3 in the **Online Supplementary Document**.

**Table 2:** Description of treatment-seeking behaviour by mothers of under-three-year-old children with symptoms of ARI in the two weeks preceding the survey interview*

	Overall† n = 17 452	Urban† n = 4047 (23.2%)	Rural† n = 13 405 (76.8%)

**Place where first sought advice or treatment**			
None	2088 (12.0%)	406 (10.0%)	1682 (12.5%)
Public	3172 (18.2%)	759 (18.7%)	2414 (18.0%)
Private	8116 (46.5%)	2030 (50.2%)	6086 (45.4%)
Pharmacy/unregulated	1186 (6.8%)	189 (4.7%)	997 (7.4%)
Missing	2890 (16.6%)	664 (16.4%)	2226 (16.6%)
**Days after onset of symptoms when treatment started**			
Mean (±SD)	1.2 (±1.2)	1.0 (±1.0)	1.3 (±1.3)
Missing	4989 (28.6%)	1073 (26.5%)	3917 (29.2%)
**Amount given to eat during ARI episode**			
No food	1738 (10.0%)	389 (9.6%)	1349 (10.1%)
Less	8899 (51.0%)	2126 (52.5%)	6773 (50.5%)
Same	3529 (20.2%)	792 (19.6%)	2737 (20.4%)
More	342 (2.0%)	63 (1.5%)	279 (2.1%)
Missing	2943 (16.9%)	677 (16.7%)	2267 (16.9%)
**Amount given to drink during ARI episode**			
No drink	766 (4.4%)	186 (4.6%)	579 (4.3%)
Less	9263 (53.1%)	2204 (54.5%)	7059 (52.7%)
Same	4031 (23.1%)	903 (22.3%)	3128 (23.3%)
More	460 (2.6%)	80 (2.0%)	380 (2.8%)
Missing	2932 (16.8%)	674 (16.7%)	2258 (16.8%)

ARI – acute respiratory infection, SD – standard deviation

*NFHS-4 and NFHS-5; n = 17 452.

†Frequency (%).

### Prevalence of antibiotic consumption in India by zones

**Figure 2:**
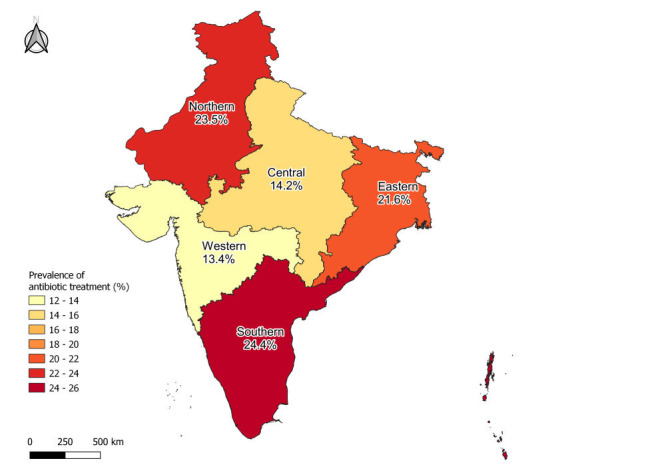
Prevalence of antibiotic treatment for under-three-year-old children with symptoms of ARI in the previous two weeks stratified by zone; NFHS-4 and NFHS-5 (n = 17 452).

To explore regional differences within India, we estimated the prevalence of children with ARI symptoms who were given antibiotics in the six different administrative zones of the country (**Figure 2**). The prevalence of antibiotic treatment ranged from 13.4 to 24.4% across zones. The zones with the highest prevalence were the Southern (24.4%) and Northern (23.5%) zones, which correspond to more urbanised and predominately rural areas, respectively. Alternatively, the lowest prevalence was found in Western (13.4%) and Central India (14.2%). The individual prevalence estimates and their 95% confidence intervals for each zone are shown in Figure S1 in the **Online Supplementary Document**.

Additionally, we estimated the prevalence of antibiotic treatment across individual states and union territories (Figures S2 and S3 in the **Online Supplementary Document**). However, the number of ARI cases in U3 children in some of the smaller, predominately rural territories was very low, resulting in imprecise estimates. The number of ARI cases and the prevalence for each zone and territory are presented in supplementary Table S4 in the **Online Supplementary Document**.

### Factors associated with antibiotic treatment

The results from the logistic regression models are shown in **Table 3**. Some sociodemographic characteristics were associated with antibiotic use across all models. For example, the prevalence of antibiotic treatment was 11.7 (95% CI = 6.3-16.7) percentage-points higher among families from religious traditions other than Hinduism or Islam (mainly Christianism, Sikhism, Buddhism and others) compared to Hindus. Additionally, antibiotic treatment was 5.8 (95% CI = 1.7-9.9) percentage-points higher among children whose mothers attained secondary or higher levels of education compared to those without any formal education.

**Table 3:** Prevalence differences for the adjusted association between potential predictors and antibiotic treatment among under-three-year-old children with symptoms of ARI from multivariable logistic regression models*

		Model 1 (n = 16 160)		Model 2 (n = 14 725)		Model 3 (n = 13 420)		Model 4 (n = 11 053)	
	**Received antibiotic**† **n = 3266 (18.7%)**	**PD**	**95% CI**	**PD**	**95% CI**	**PD**	**95% CI**	**PD**	**95% CI**
**Urban/rural**									
Urban	860.2 (26.3%)	—‡	—‡	—‡	—‡	—‡	—‡	—‡	—‡
Rural	2405.4 (73.3%)	-1.1	-3.9, 1.7	-0.6	-3.6, 2.3	-0.2	-3.3, 2.9	-0.5	-4.0, 3.1
**Wealth index**									
Poorest	800.2 (24.5%)	—‡	—‡	—‡	—‡	—‡	—‡	—‡	—‡
Poorer	757.0 (23.2%)	0.9	-1.4, 3.3	-0.9	-3.6, 1.7	-1.1	-3.9, 1.6	-2.5	-5.7, 0.7
Middle	581.5 (17.8%)	-0.9	-3.3, 1.6	-2.6	-5.8, 0.5	-2.1	-5.4, 1.2	-3.3	-7.2, 0.6
Richer	635.4 (19.5%)	3.0	0.1, 6.0	0.2	-3.7, 4.1	0.5	-3.6, 4.6	-1.0	-5.9, 3.8
Richest	491.4 (15.0%)	3.0	-0.8, 6.9	0.2	-4.7, 5.1	-0.6	-5.6, 4.4	-2.1	-8.1, 3.9
**Religion**									
Hindu	2485.1 (76.1%)	—‡	—‡	—‡	—‡	—‡	—‡	—‡	—‡
Muslim	584.4 (17.9%)	0.7	-2.0, 3.4	1.0	-1.8, 3.8	1.0	-2.0, 4.0	0.4	-3.0, 3.8
Others	196.0 (6.0%)	11.8	7.3-16.1	11.2	6.5-15.6	10.5	5.6-15.1	11.7	6.3-16.7
**Caste/tribe**									
None	591.4 (19.1%)	—‡	—‡	—‡	—‡	—‡	—‡	—‡	—‡
Schedule tribe	696.5 (22.5%)	-2.40	-5.8, 0.9	-3.10	-6.6, 0.2	-3.30	-7.0, 0.2	-2.7	-7.1, 1.6
Schedule caste	248.9 (8.0%)	-1.00	-3.8, 1.7	-1.30	-4.2, 1.5	-1.40	-4.3, 1.6	-2.0	-5.4, 1.5
Other backward classes	1556.5 (50.3%)	1.90	-0.6, 4.5	1.50	-1.1, 4.1	1.40	-1.2, 4.1	2.0	-1.1, 5.1
**Mother’s age (years)**	25.9 (±4.8)	0.2	0.0-0.3	0.1	-0.1, 0.3	-0.1	-0.3, 0.2	0.0	-0.3, 0.3
**Mother’s educational level**									
No education	661.5 (20.3%)	—‡	—‡	—‡	—‡	—‡	—‡	—‡	—‡
At least some primary	451.0 (13.8%)	1.9	-0.8, 4.7	2.2	-0.6, 5.0	2.0	-0.9, 4.9	1.6	-1.7, 5.0
Incomplete secondary	1476.0 (45.2%)	2.2	0.0-4.4	2.7	0.4-5.1	2.7	0.2-5.2	2.9	-0.1, 5.8
Secondary or higher	677.0 (20.7%)	4.2	1.3-7.3	3.8	0.5-7.0	4.9	1.4-8.4	5.8	1.7-9.9
**Household size**	6.3 (±2.7)			-0.3	-0.6, 0.0	-0.3	-0.6, 0.0	-0.3	-0.7, 0.1
**Any deceased children**									
No	2931.9 (89.8%)			—‡	—	—‡	—‡	—‡	—‡
Yes	333.6 (10.2%)			2.6	-0.5, 5.8	1.2	-2.1, 4.6	1.0	-2.8, 4.7
**Type of cooking fuel**									
Clean	1269.7 (41.4%)			—‡	—‡	—‡	—‡	—‡	—‡
Unclean	1797.4 (58.6%)			0.4	-2, 2.8	0.3	-2.1, 2.8	-0.4	-3.3, 2.5
**Source of drinking water**									
Improved	2940.4 (96.0%)			—‡	—‡	—‡	—‡	—‡	—‡
Unimproved	123.6 (4.0%)			-1.1	-4.7, 2.5	-1.3	-5, 2.4	-1.4	-5.8, 3.1
**Toilet facility**									
Improved	1898.8 (62.1%)			—‡	—‡	—‡	—‡	—‡	—‡
Unimproved	1159.7 (37.9%)			-1.3	-3.5, 0.8	-1.0	-3.2, 1.3	-2.2	-4.8, 0.5
**Soap for handwashing**									
No	1119.9 (34.9%)			—‡	—‡	—‡	—‡	—‡	—‡
Yes	2093.5 (65.1%)			0.7	-1.3, 2.7	0.5	-1.7, 2.6	0.5	-1.9, 2.9
**Media accessibility**									
Not at all	810.5 (24.8%)			—‡	—‡	—‡	—‡	—‡	—‡
Less than once a week	479.0 (14.7%)			-1.2	-3.8, 1.4	-1.0	-3.7, 1.8	0.1	-3.3, 3.4
At least once a week	895.4 (27.4%)			-0.7	-3.2, 1.8	-1.2	-3.8, 1.4	0.6	-2.7, 3.9
Almost every day	1080.5 (33.1%)			5.3	2.2-8.4	4.8	1.6-8.0	0.1	-3.4, 3.7
**Below poverty line card**									
No	1969.9 (60.5%)			—‡	—‡	—‡	—‡	—‡	—‡
Yes	1288.4 (39.5%)			0.4	-1.6, 2.3	0.1	-1.8, 2.1	-0.4	-2.7, 2.0
**Smoke exposure**									
Never	1779.0 (54.5%)			—‡	—‡	—‡	—‡	—‡	—‡
Daily	963.2 (29.5%)			-1.1	-3.2, 0.9	-1.1	-3.2, 1.0	-0.7	-3.1, 1.8
Weekly or less	523.3 (16.0%)			-1.7	-4, 0.7	-1.3	-3.8, 1.2	-1.1	-4.1, 1.8
**Sex**									
Male	1789.0 (54.8%)					—‡	—‡	—‡	—‡
Female	1476.5 (45.2%)					0.0	-1.9, 1.8	1.0	-1.2, 3.1
**Age (months)**	17.6 (±9.5)					0.1	0.0,-0.2	0.1	-0.1, 0.2
**Birth order**	2.2 (±1.4)					1.2	0.2-2.3	1.1	-0.1, 2.3
**Place of delivery**									
Public	1918.3 (58.7%)					—‡	—‡	—‡	—‡
Private	895.9 (27.4%)					0.8	-1.5, 3	-0.3	-2.9, 2.3
Home	437.2 (13.4%)					1.1	-1.6, 3.8	0.5	-2.6, 3.6
Other/NGO	14.1 (0.4%)					-4.5	-13.6, 4.5	-3.4	-15.3, 8.6
**Stunting (height/age)**									
Not stunted	2006.3 (66.3%)					—‡	—‡	—‡	—‡
Moderately stunted	570.0 (18.8%)					-1.1	-3.3, 1.2	-1.5	-4.1, 1.1
Severely stunted	448.3 (14.8%)					-1.8	-4.5, 0.8	-2.4	-5.5, 0.6
**Wasting (weight/height)**									
Not wasted	2293.1 (76.2%)					—‡	—‡	—‡	—‡
Moderately wasted	457.2 (15.2%)					2.0	-0.6, 4.7	1.7	-1.2, 4.7
Severely wasted	260.2 (8.6%)					2.5	-1.4, 6.4	3.2	-1.3, 7.8
**Has vaccination card**									
No card	260.3 (8.0%)					—‡	—‡	—‡	—‡
Yes, seen	2,628.1 (80.5%)					4.1	0.9-7.2	4.3	0.5-8.0
Yes, not seen	377.1 (11.5%)					-0.9	-4.5, 2.7	-2.2	-6.4, 2.1
**Fully immunised**									
No	1481.7 (45.4%)					—‡	—‡	—‡	—‡
Yes	1782.3 (54.6%)					3.3	1.1-5.5	2.4	-0.1, 4.9
**Problems accessing health care**	4.8 (±0.05)							-0.5	-1.0, -0.1
**Health workers visit in last 3 months**									
No	1230.6 (37.7%)							—‡	—‡
Yes	2034.9 (62.3%)							1.8	-0.6, 4.2
**Covered by health insurance**									
No	2592.0 (79.4%)							—‡	—‡
Yes	673.5 (20.6%)							5.1	2.0-8.1
**Anganwadi or ICDS benefits**									
No	944.0 (28.9%)							—‡	—‡
Yes	2321.5 (71.1%)							0.9	-2.0, 3.7
**Drugs for intestinal parasites (last 6 months)**									
No	2057.4 (63.2%)							—‡	—‡
Yes	1195.5 (36.8%)							3.3	0.5-6.0
**Vitamin A supplementation (last 6 months)**									
No	1292.0 (39.8%)							—‡	—‡
Yes	1955.2 (60.2%)							0.4	-2.0, 2.8
**Iron supplementation (last 7 days)**									
No	2251.2 (69.1%)							—‡	—‡
Yes	1006.0 (30.9%)							-2.0	-4.6, 0.7
**Place where first sought advice or treatment**									
No treatment	161.1 (4.9%)							—‡	—‡
Public	770.6 (23.6%)							14.9	12.0-17.9
Private	2063.4 (63.2%)							16.1	13.8-18.8
Pharmacy/unregulated	270.4 (8.3%)							14.9	10.8-19.2

ARI – acute respiratory infections, ICDS – integrated child development services, PD – prevalence difference, CI – confidence interval, NGO – non-governmental organisation

*NFHS-4 and NFHS-5; n = 17 452.

†Mean (± standard deviation (SD)) or frequency (%). The percentage corresponds to the distribution of the different categories among those that received antibiotic.

‡The categories used as reference for the model are represented by —.

Some sociodemographic factors were associated with antibiotic use in models controlling for socio-demographic characteristics, but associations were attenuated in the final model that included health care-related factors. This includes, for example, those in the second highest (richer) quintile of the wealth index, families who access media every day, and children who were fully immunised.

Overall, antibiotic use for the treatment of ARI symptoms was associated with factors that indicate greater access and interactions with the health care system. For instance, per each reported problem accessing health care (median 6.0; interquartile range (IQR) = 3.0-8.0), the prevalence of antibiotic treatment was 0.5 (95% CI = 0.1-1.0) percentage-points lower. Conversely, the prevalence of antibiotic treatment was 5.1 (95% CI = 2.0-8.1) percentage-points higher among children covered by any health insurance compared to those who were not covered. Similarly, the prevalence of antibiotic treatment was 4.3 (95% CI = 0.5-8.0) percentage-points higher among children who had a vaccination card and showed it to the interviewer than those without a card and 3.3 (95% CI = 0.5-6.0) percentage-points higher among those who received drugs for intestinal parasites in the previous six months than those who did not.

The places where mothers sought first treatment or advice were the strongest predictors of antibiotic use; those seeking treatment in private health care, public institutions, and pharmacy or unregulated sources were 16.1 (95% CI = 13.8-18.8), 14.9 (95% CI = 12.0-17.9), and 14.9 (95% CI = 10.8-19.2) percentage-points more likely to receive antibiotic treatment for their children than mothers who did not seek any advice or treatment.

## DISCUSSION

Our study, based on the latest nationally representative health survey data in India, showed that 18.7% (95% CI = 17.8-19.6) of under-three-year-old children with symptoms of ARI in the prior two weeks received antibiotics as treatment. Antibiotic use was higher among children in the Southern, Northern, and Eastern zones, but was similar between urban and rural populations. Multivariable regression analyses of the potential predictors of receiving antibiotics for ARI treatment showed positive associations between indicators of socioeconomic position, such as mother’s educational level, and antibiotic use. Access to and use of health care, as indicated by health insurance coverage, reporting fewer problems accessing health care, and seeking advice or treatment for the child’s symptoms of ARI, irrespective of the source of care, was strongly associated with the prevalence of antibiotic use. Although formal mediation analyses are necessary, socioeconomic gradients in use may be partly explained by differences in accessibility to and use of health care.

To our knowledge, this is the first study to assess antibiotic use for the treatment of ARI among children in India using community-level data. Our estimated prevalence of 18.7% (95% CI = 17.8-19.6) was substantially lower compared to estimates in U5 children in neighboring countries, including an estimated 39% prevalence in Bangladesh and 40.7% in Nepal [37,38]. Our estimates are also far lower than the >80% prevalence from eight LMICs (Haiti, Kenya, Malawi, Namibia, Nepal, Senegal, Tanzania and Uganda) [49]. The wide range of prevalence estimates for ARI-related antibiotic treatment in children may be related to heterogeneity in definitions of ARI episodes and study populations. Additionally, most extant research has used hospital-based samples, in contrast to the community-based target population of the NFHS, and may have selected for more symptomatic cases who are already interacting with the health care system. Indeed, a cohort study in a rural hospital setting in Madhya Pradesh, India estimated a prevalence of 46% in U5 children [50].

In contrast to previous studies in India and neighboring countries, where it was found that children with ARI were more likely to be treated with antibiotics in rural areas [37,38,51], we did not find marked differences between urban and rural populations. This could be explained by the efforts of the government to strengthen primary care in rural India [52], where traditional healers and informal health care providers were more prevalent than in urban areas, and prescribed more antibiotics [50]. Also, this is consistent with our results, where women in both urban and rural settings were 33 most likely to seek initial treatment in private health care settings, although women in rural areas were slightly more likely than their urban counterparts to seek care from pharmacies or unregulated facilities. Our regional analysis shows a higher prevalence of antibiotic treatment in Southern and Northern India and the state-level results shown in the Figures S2 and S3 in the **Online Supplementary Document** demonstrated a higher prevalence in Kerala and Jammu and Kashmir. Despite the efforts from the Indian government to regulate the OTC antibiotic sales through the Schedule H1 in 2013, seeking advice or treatment at pharmacies, informal health care providers, traditional healers or other sources is associated with an increased prevalence of antibiotic treatment. This suggests that despite government efforts to limit antibiotic prescriptions to formal health care providers, illegal antibiotic prescriptions and OTC sales continue, as shown in previous studies [23,24,53].

One of the main challenges of AMR in India is the antimicrobial stewardship of human and animal health professionals [54]. In line with our results, enhancing educational programmes and antimicrobial stewardship through all actors involved, from doctors to patients at public, private, or unregulated facilities, could have a profound impact on the reduction of antibiotic consumption in children with ARI symptoms. These efforts should be targeted, for example to zones (i.e. Southern and Northern India), health care providers, and subgroups (i.e. socioeconomically advantaged) who are more likely to receive antibiotic treatments. Some pilot antimicrobial stewardship strategies at the hospital level have proven to be effective on a low-budget intervention and could be replicated in other areas of the country [55,56].

There are several limitations to this study. First, there is no consensus in the field regarding the definition of ARI symptoms. Our definition of ARI – cough accompanied by short, rapid or difficult breathing – covers a broader range of milder ARIs compared to cases where ARI is defined as a cough accompanied by short, rapid, or difficult breathing due to a problem in the chest, which relates more to LRTIs [1]. Therefore, we believe our ARI definition provides a more relevant indicator of unsuitable antibiotic use. However, ARI cases were identified based on self-reports and not validated by a clinician or any diagnostic tool. Nonetheless, this syndromic approach has been applied previously to identify ARI cases from community-based surveys in India [57]. There may have been recall bias when assessing child illnesses such as ARIs, with prior research suggesting that a two-week recall period underestimates the prevalence of diarrheal illness compared to one-week recall [58]. However, other research looking at diagnosis of pneumonia has shown only modest differences in results when varying the recall period [59]. Error in the assessment of ARI could affect selection into our sample (e.g. some ARI “cases” were incorrectly classified as “negative”), which could bias results. For example, if there are other characteristics that influence the degree of error in the measurement of ARI that also affect the treatment received (i.e. antibiotic use), then our estimates of the prevalence of antibiotic use may be biased. However, it is less plausible that our analyses of the predictors of antibiotic use would be affected by this type of selection. With respect to measuring antibiotic use, some research suggests that household surveys provide fairly accurate measurement of treatments given to children reporting a fever in the two weeks prior to their survey [60], suggesting there is less error in the measurement of treatments given vis-à-vis diagnosis. If error in the measurement of antibiotic consumption was random this may bias associations between predictors of use (e.g. covered by health insurance) and reported consumption toward the null. However, if the error was differential, associations could be over- or underestimated, depending on the nature of the misclassification.

Second, non-response and selection bias were minimised by applying sampling weights; however, missing covariate information reduced sample sizes available for complete case analyses, introducing imprecision, and may have biased measures of association if data were not missing completely at random. Third, due to changes between the NFHS-4 and NFHS-5, specifically to the age range of children included in the Child Immunizations and Health module, we limited our sample to children under 36 months of age. This could limit the comparison of our results with others, which typically include children less than 60 months [37,38]. Fourth, it is important to acknowledge that the statistical analyses were descriptive in nature and aimed to identify predictor variables rather than develop a prediction model or assess the causal effect of any particular exposure or treatment. Finally, we lacked information on the etiology of ARI symptoms, which limits our ability to determine the proportion of antibiotic misuse. Nevertheless, microbiological diagnosis of ARI is not routinely done in India and it is believed that more than 80% of ARI episodes in children are due to viral infections [12].

## CONCLUSIONS

In summary, our study estimated a national prevalence of antibiotic use of 18.7% (95% CI = 17.8-19.6) among U3 children with symptoms of ARI in the prior two weeks, with a higher prevalence observed in the Southern and Northern zones of India. We found that children from households with better health care access, including health insurance coverage, and those who sought care from public, private, or unregulated health care services, compared to not seeking care, were more likely to receive antibiotics. These findings emphasise the need for targeted interventions to enhance antimicrobial stewardship and for the development of stricter policies regarding over-the-counter sales of antibiotics. Further research, including standardised patient studies, is warranted to assess not only antibiotic use but also inappropriate usage.

## Additional material


Online Supplementary Document
