# Supplementary material for: Patterns of antibiotic use for acute respiratory infections in under-three-year-old children in India: A cross-sectional study
Source: J Glob Health. 2023 Dec 22;13:04159. doi: 10.7189/jogh.13.04159 (PMC10740384; doi:10.7189/jogh.13.04159)
Supplement: Online Supplementary Document [file jogh-13-04159-s001.pdf]

Supplementary material

Patterns of antibiotic use for acute respiratory infections in under-three-year-old children in India: a cross-sectional study

Hector G. Dorta[1], Arijit Nandi[1]

[1]Institute for Health and Social Policy & Department of Epidemiology, Biostatistics, and Occupational Health, McGill University, Montreal, Canada

1. Definition of predictor variables

Table S1. Predictor variables definitions.

**Demographic variables**	
Place of residence (urban/rural)	According to the Indian census definition: a habitation is declared urban if it has a municipality, municipal corporation, cantonment board or notified area committee or if it has a minimum population of 5,000; at least 75% of the male working population is engaged in non-agricultural pursuits; and the population density is at least 400 people/km$^{2.}$ Anything else is considered a rural area, which its basic unit it is the village.
Wealth index	The wealth index was calculated using easy-to-collect data on a household's ownership of selected assets (consumer items and housing characteristics). Generated with a statistical procedure (principal components analysis) the wealth index places individual households on a continuous scale of relative wealth, NFHS categorizes all surveyed households into five wealth quintiles.
Religion	Respondents were asked about their religion and answers were grouped as (1) Hindu, (2) Muslim, and (3) Others including all possible other options.
Caste/Tribe	Whether the respondents belong to a scheduled caste, scheduled tribe, other backward class, or none of them.
Mother's age	Age of the mother at the time of the interview.
Mother's educational level	Classified as no education, at least some primary, incomplete secondary, and secondary or higher education.
Zones	The states of India were grouped according to the six Advisory Council zones.<br>– Northern: Chandigarh, Delhi, Haryana, Himachal Pradesh, Jammu and Kashmir, Ladakh, Punjab, and Rajasthan.<br>– Northeastern: Assam, Arunachal Pradesh, Manipur, Meghalaya, Mizoram, Nagaland, Tripura, and Sikkim.<br>– Central: Chhattisgarh, Madhya Pradesh, Uttarakhand, and Uttar Pradesh.<br>– Eastern: Bihar, Jharkhand, Odisha, and West Bengal<br>– Western: Dadra and Nagar Haveli and Daman and Diu, Goa, Gujarat, and Maharashtra.<br>– Southern: Andaman and Nicobar Islands, Andhra Pradesh, Karnataka, Kerala, Lakshadweep, Puducherry, Tamil Nadu, and Telangana.
**Household variables**	
Household size	Number of people living in the same household, Answers including more than 10 people were regrouped as one single category.
Children deceased	Whether any previous son or daughter has passed away.
Media accessibility (TV or radio)	Composite variable that registers whether the respondent reads, listens to the radio, or watches television almost every day, at least once a week, less than once a week, or not at all.
Source of drinking water	Categorized as improved (including piped into dwelling, piped to yard/plot, public tap/standpipe, tube well water, tube well or borehole, protected well, protected spring, rainwater, tanker truck, cart with a small tank and bottled water, and community reverse osmosis plant) or

	unimproved (unprotected well, surface water, unprotected spring, river/dam/lake/ponds/stream/canal/irrigation channel). Other answers were recoded as missing observations.
Toilet facility	Categorized as improved (flush toilet, flush to piped sewer system, flush to septic tank, flush to pit latrine, flush to pit latrine, pit toilet latrine, ventilated improved pit latrine, pit latrine with slab and composting toilet) or unimproved (dry toilet, flush to somewhere else, flush to don't know where, pit latrine without slab/open pit, no facility, no facility/bush/field. Other answers were recoded as missing observations.
Type of cooking fuel	Categorized as clean (gas, liquefied petroleum gas/natural gas, and electricity) or unclean (kerosene, coal/lignite, charcoal, wood, straw/shrubs/grass, agricultural crop waste, and dung cakes). Other answers were recoded as missing observations.
Below Poverty Line (BPL) card	Whether the family possess or not a BPL ration card. The threshold is specified by each state government, BPL families receive 10kg to 20kg of food grains per family per month at 50% of the economic cost.
Hand hygiene	Presence of soap or detergent at the place where the household washes their hands.
Smoke exposure	Frequency of household members smoking inside the house, Categorized as never, daily, weekly, or less.
**Child-related variables**	
Age	Age of the child in months at the time of the interview. Calculated from century month code (CMC) and century day code (CDC) for NFHS-4 and NFHS-5, respectively.
Sex	Gender of the children assessed as male or female.
Birth order	Order in which the child was born.
Place of delivery	Place where the child was delivered. Categorized as public (including government/municipal hospital, government dispensary, union health complex, union health and family welfare center, community health center/rural hospital/block public health center, and other public health centers or sub-center facilities), private (including private hospital, clinic, maternity home, or other private sector health facility), at home (including respondent's home, parents' home, or other home) and NGO/Other health facilities.
Stunting	Used as a proxy for chronic undernutrition. Defined as per the new WHO Child Growth Standards as height-for-age. Classified as severely stunted if > -3.0 or moderately stunted if > -2.0 below the mean or not stunted.
Wasting	Used as a proxy for acute malnutrition. Defined as per the new WHO Child Growth Standards as weight-for-height. Classified as severely wasted if > -3.0 or moderately wasted if > -2.0 below the mean or not wasted.
**Healthcare-related variables**	
Access to healthcare	Composite variable scoring the number of problems that the mother reported when getting medical help. It ranges from 0 to 8. Each of the following problems scores 1 point: getting permission to go, getting money needed for the treatment, distance to the health facility, having to take transport, not wanting to go alone, concern there is no female health provider, concern there is no provider, concern there are no drugs available.

Health worker visit in last 3 months	Whether the respondent had met with an Anganwadi worker, Asha, or other community health worker in the last 3 months before the interview.
Health insurance	Whether the respondent is covered by any health scheme or any health insurance.
Vaccination card	Whether the child has a vaccination card, and the mother was able to show it to the interviewer. Categorized as: no card; yes, seen, and yes, not seen.
Fully immunized	Whether the child has received three doses of DPT vaccine, three doses of polio vaccine, a measles vaccine, and a BCG vaccine.
Anganwadi or ICDS benefits	Whether the child received benefits from Anganwadi/ICDS center in the last 12 months before the interview.
Medication for intestinal parasites	Whether the child received drugs for intestinal parasites in the last 6 months prior to the interview.
Iron supplementation	Whether the child received iron supplementation in the last 7 days prior to the interview.
Vitamin A supplementation	Whether the child received vitamin A supplementation in the last 6 months prior to the interview.
Place where first sought advice or treatment	Place where the mother first sought advice or treatment for ARI symptoms. Categorized as no treatment, public (including government/municipal hospital, government dispensary, union health and family welfare center, community health center/rural hospital/block public health center, additional public health centers or sub-center facilities, government mobile clinic, camp, NGO or trust hospital/clinic, Anganwadi/ICDS center, Asha or other public health facilities), private (including private hospital, clinic, paramedic or other private sector health facility) and pharmacy/unregulated (including pharmacy/drugstore, public or private traditional Ayush treatment, traditional healers, shop, friend/relative or other unauthorized sources).
Days after treatment	Number of days after the onset of cough symptoms until treatment or advice was sought. Categorized as same day, one, two, three, or four or more days.
Amount offered to drink	Amount offered to drink during the ARI episode. Categorized as no drink, less, same, or more than usual.
Amount offered to eat	Amount offered to eat during the ARI episode. Categorized as no drink, less, same, or more than usual.

2. Unweighted descriptive analysis

Table S2. Distribution of unweighted sociodemographic characteristics, household variables, child-related variables, and healthcare variables of under-three-year-old children with symptoms of ARI in the two weeks preceding the survey interview; NFHS-4 and NFHS-5; N = 16,972.

	**Total (N=16972)**[1]	**Urban (N=3402)**[1]	**Rural (N=13570)**[1]
**Outcome variable**			
**Were given antibiotics**			
No	13,623 (80.3%)	2656 (78.1%)	10,967 (80.8%)
Yes	3349 (19.7%)	746 (21.9%)	2603 (19.2%)
**Sociodemographic variables**			
**Wealth index**			
Poorest	4729 (27.9%)	198 (5.8%)	4531 (33.4%)
Poorer	4210 (24.8%)	390 (11.5%)	3820 (28.2%)
Middle	3366 (19.8%)	717 (21.1%)	2649 (19.5%)
Richer	2681 (15.8%)	1003 (29.5%)	1678 (12.4%)
Richest	1986 (11.7%)	1094 (32.2%)	892 (6.6%)
**Religion**			
Hindu	12,665 (74.6%)	2332 (68.5%)	10,333 (76.1%)
Muslim	2768 (16.3%)	778 (22.9%)	1990 (14.7%)
Others	1539 (9.1%)	292 (8.6%)	1247 (9.2%)
**Caste/Tribe**			
Scheduled caste	3699 (21.8%)	707 (20.8%)	2992 (22.0%)
Schedule tribe	2692 (15.9%)	285 (8.4%)	2407 (17.7%)
Other Backward Class	6912 (40.7%)	1493 (43.9%)	5419 (39.9%)
None	2857 (16.8%)	773 (22.7%)	2084 (15.4%)
Missing	812 (4.8%)	144 (4.2%)	668 (4.9%)
**Mother's age (years)**			
Mean (± SD)	26.0 (± 4.8)	26.4 (± 4.6)	25.8 (± 4.8)
**Mother's educational level**			
No education	4113 (24.2%)	478 (14.1%)	3635 (26.8%)
At least some primary	2460 (14.5%)	421 (12.4%)	2039 (15.0%)
Incomplete secondary	7549 (44.5%)	1500 (44.1%)	6049 (44.6%)
Secondary or higher	2850 (16.8%)	1003 (29.5%)	1847 (13.6%)
**Household variables**			
**Household size**			
Mean (± SD)	6.5 (± 2.8)	6.5 (± 2.9)	6.5 (± 2.8)
**Prior children deceased**			
No	15,329 (90.3%)	3179 (93.4%)	12,150 (89.5%)
Yes	1643 (9.7%)	223 (6.6%)	1420 (10.5%)
**Type of cooking fuel**			
Clean	5404 (31.8%)	2326 (68.4%)	3078 (22.7%)
Unclean	10,514 (61.9%)	865 (25.4%)	9649 (71.1%)
Missing	1054 (6.2%)	211 (6.2%)	843 (6.2%)
**Source of drinking water**			
Improved	14,684 (86.5%)	3082 (90.6%)	11,602 (85.5%)
Unimproved	1196 (7.0%)	96 (2.8%)	1100 (8.1%)
Missing	1092 (6.4%)	224 (6.6%)	868 (6.4%)
**Toilet facility**			
Improved	9274 (54.6%)	2658 (78.1%)	6616 (48.8%)
Unimproved	6614 (39.0%)	519 (15.3%)	6095 (44.9%)
Missing	1084 (6.4%)	225 (6.6%)	859 (6.3%)

	Total (N=16972)[1]	Urban (N=3402)[1]	Rural (N=13570)[1]
**Outcome variable**			
No	5976 (35.2%)	615 (18.1%)	5361 (39.5%)
Yes	10,647 (62.7%)	2742 (80.6%)	7905 (58.3%)
Missing	349 (2.1%)	45 (1.3%)	304 (2.2%)
**Media accessibility**			
Not at all	4731 (27.9%)	360 (10.6%)	4371 (32.2%)
Less than once a week	2961 (17.4%)	450 (13.2%)	2511 (18.5%)
At least once a week	4768 (28.1%)	1139 (33.5%)	3629 (26.7%)
Almost every day	4512 (26.6%)	1453 (42.7%)	3059 (22.5%)
**Below Poverty Line card**			
No	9815 (57.8%)	2425 (71.3%)	7390 (54.5%)
Yes	7118 (41.9%)	970 (28.5%)	6148 (45.3%)
Missing	39 (0.2%)	7 (0.2%)	32 (0.2%)
**Smoke exposure**			
Never	8054 (47.5%)	1849 (54.4%)	6205 (45.7%)
Daily	5627 (33.2%)	964 (28.3%)	4663 (34.4%)
Weekly or less	3291 (19.4%)	589 (17.3%)	2702 (19.9%)
**Age (months)**			
Mean (± SD)	17.0 (± 9.7)	17.3 (± 9.7)	16.9 (± 9.7)
**Sex**			
Male	9291 (54.7%)	1854 (54.5%)	7437 (54.8%)
Female	7681 (45.3%)	1548 (45.5%)	6133 (45.2%)
**Birth order**	2.19 (± 1.40)	1.98 (± 1.23)	2.24 (± 1.43)
**Place of delivery**			
Public	10,402 (61.3%)	1819 (53.5%)	8583 (63.2%)
Private	3618 (21.3%)	1204 (35.4%)	2414 (17.8%)
Home	2841 (16.7%)	341 (10.0%)	2500 (18.4%)
Other/NGO	111 (0.7%)	38 (1.1%)	73 (0.5%)
**Stunting (height/age)**			
Not stunted	10,091 (59.5%)	2188 (64.3%)	7903 (58.2%)
Moderately stunted	3040 (17.9%)	519 (15.3%)	2521 (18.6%)
Severely stunted	2550 (15.0%)	411 (12.1%)	2139 (15.8%)
Missing	1291 (7.6%)	284 (8.3%)	1007 (7.4%)
**Wasting (weight/height)**			
Not wasted	12,233 (72.1%)	2498 (73.4%)	9735 (71.7%)
Moderately wasted	2077 (12.2%)	359 (10.6%)	1718 (12.7%)
Severely wasted	1277 (7.5%)	236 (6.9%)	1041 (7.7%)
Missing	1385 (78.2%)	309 (9.1%)	1076 (7.9%)
**Healthcare variables**			
**Problems accessing healthcare**			
Mean (± SD)	5.2 (± 2.9)	4.3 (± 2.8)	5.5 (± 2.5)
**Health worker visit in last 3 months**			
No	6612 (39.0%)	1615 (47.5%)	4997 (36.8%)
Yes	10,360 (61.0%)	1787 (52.5%)	8573 (63.2%)
**Covered by health insurance**			
No	13,723 (80.9%)	2813 (82.7%)	10,910 (80.4%)
Yes	3249 (19.1%)	589 (17.3%)	2660 (19.6%)
**Vaccination card**			
No card	1844 (10.9%)	344 (10.1%)	1500 (11.1%)
Yes, seen	12,729 (75.0%)	2596 (76.3%)	10,133 (74.7%)
Yes, not seen	2399 (14.1%)	462 (13.6%)	1937 (14.3%)

	**Total (N=16972)[1]**	**Urban (N=3402)[1]**	**Rural (N=13570)[1]**
**Outcome variable**			
No	9072 (53.5%)	1752 (51.5%)	7320 (53.9%)
Yes	7865 (46.3%)	1645 (48.4%)	6220 (45.8%)
Missing	35 (0.2%)	5 (0.1%)	30 (0.2%)
**Anganwadi or ICDS benefits**			
No	5025 (29.6%)	1406 (41.3%)	3619 (26.7%)
Yes	11,947 (70.4%)	1996 (58.7%)	9951 (73.3%)
**Drugs for intestinal parasites (last 6 months)**			
No	11,260 (66.3%)	2292 (67.4%)	8968 (66.1%)
Yes	5615 (33.1%)	1095 (32.2%)	4520 (33.3%)
Missing	97 (0.6%)	15 (0.4%)	82 (0.6%)
**Vitamin A supplementation (last 6 months)**			
No	7278 (42.9%)	1481 (43.5%)	5797 (42.7%)
Yes	9535 (56.2%)	1892 (55.6%)	7643 (56.3%)
Missing	159 (0.9%)	29 (0.9%)	130 (1.0%)
**Iron supplementation (last 7 days)**			
No	11,623 (68.5%)	2362 (69.4%)	9261 (68.2%)
Yes	5270 (31.1%)	1031 (30.3%)	4239 (31.2%)
Missing	79 (0.5%)	9 (0.3%)	70 (0.5%)

[1]Mean (± SD) or Frequency (%)

ARI: acute respiratory infection; ICDS: Integrated Child Development Services.

Table S3. Description of treatment-seeking behavior by mothers of under-three-year-old children with symptoms of ARI in the two weeks preceding the survey interview; NFHS-4 and NFHS-5; N = 16,972.

	Overall N = 16,972[1]	Urban N = 3,402 (20%)[1]	Rural N = 13,570 (80%)[1]
**Place where first sought advice or treatment**			
None	2193 (12.9%)	350 (10.3%)	1843 (13.6%)
Public	3746 (22.1%)	749 (22.0%)	2997 (22.1%)
Private	7118 (41.9%)	1578 (46.4%)	5540 (40.8%)
Pharmacy/Unregulated	1129 (6.7%)	177 (5.2%)	952 (7.0%)
Missing	2786 (16.4%)	548 (16.1%)	2238 (16.5%)
**Days after onset of symptoms when treatment started**			
Mean (± SD)	1.3 (± 1.3)	1.1 (± 1.1)	1.3 (± 1.3)
Missing	5000 (29.5%)	902 (26.5%)	4098 (30.2%)
**Amount given to eat during ARI episode**			
No food	1681 (9.9%)	346 (10.2%)	1335 (9.8%)
Less	8477 (49.9%)	1735 (51.0%)	6742 (49.7%)
Same	3620 (21.3%)	701 (20.6%)	2919 (21.5%)
More	342 (2.0%)	58 (1.7%)	284 (2.1%)
Missing	2852 (16.8%)	562 (16.5%)	2290 (16.9%)
**Amount given to drink during ARI episode**			
No drink	680 (4.0%)	136 (4.0%)	544 (4.0%)
Less	8845 (52.1%)	1815 (53.4%)	7030 (51.8%)
Same	4118 (24.3%)	798 (23.5%)	3320 (24.5%)
More	496 (2.9%)	95 (2.8%)	401 (3.0%)
Missing	2833 (16.7%)	558 (16.4%)	2275 (16.8%)

[1]Frequency (%)

ARI: acute respiratory infection

3. Regional analyses

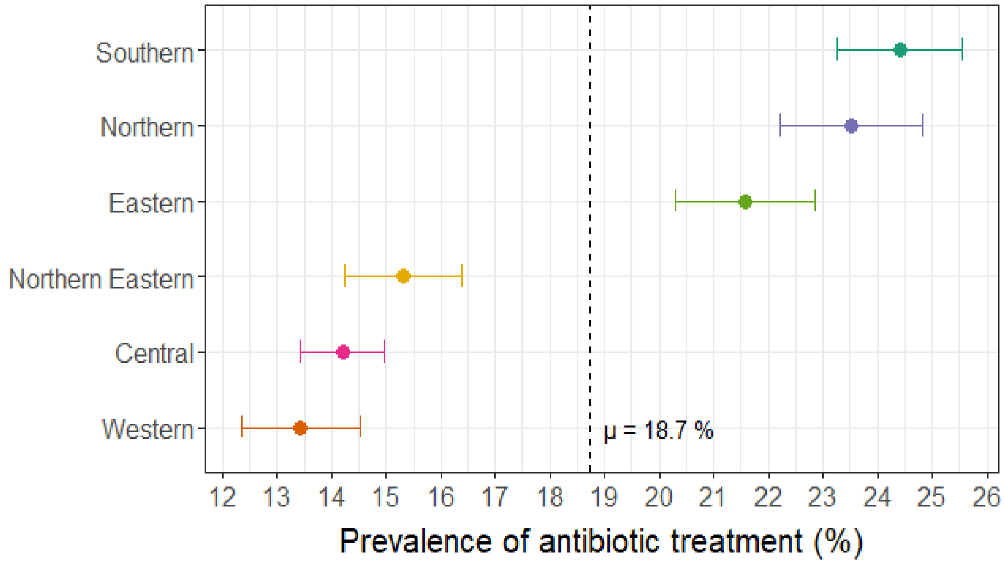

Figure S1. Prevalence of under-three-year-old children who received antibiotics to treat acute respiratory infection symptoms according to the zone of India. Bars correspond to 95% confidence intervals and the dashed line represents the mean value.

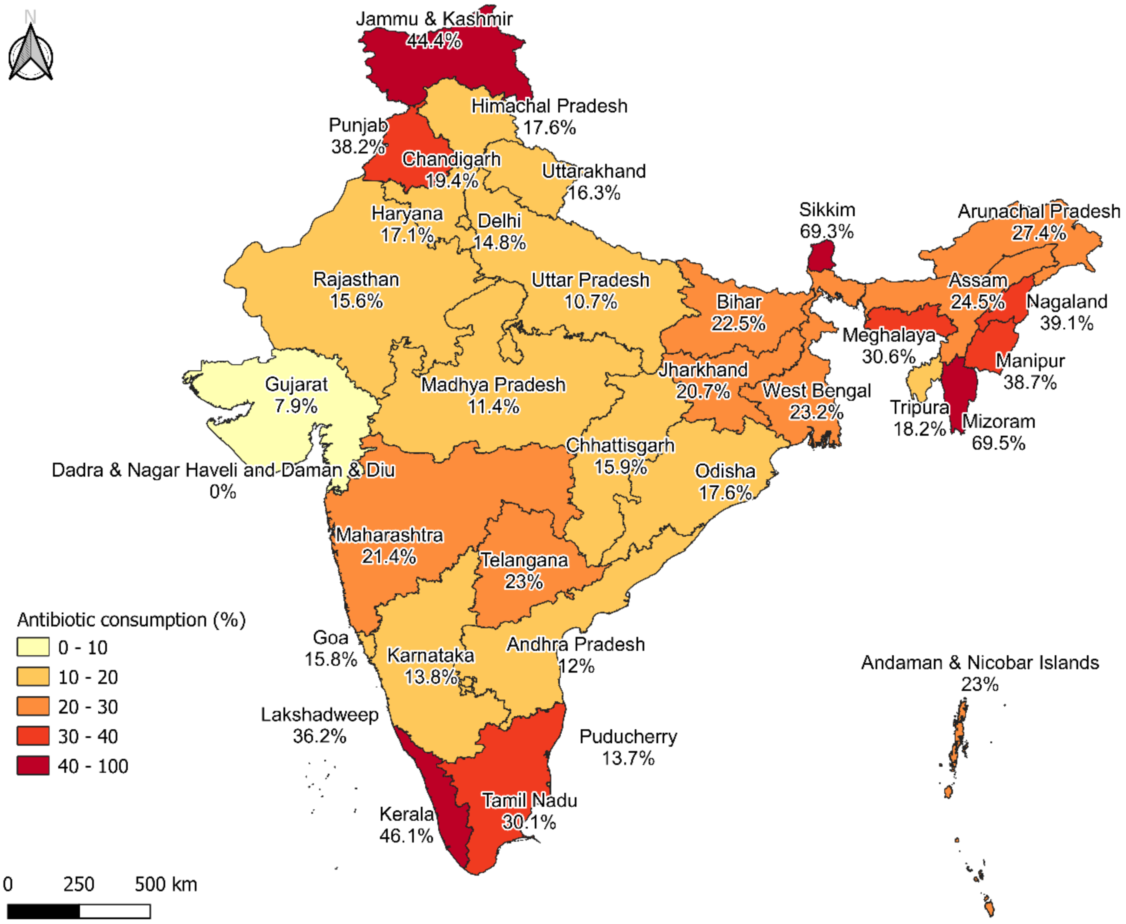

Figure S2. Prevalence of antibiotic treatment for under-three-year-old Indian children with symptoms of acute respiratory infections in the previous two weeks, stratified by zone; NFHS-4 and NFHS-5; N = 17,452.

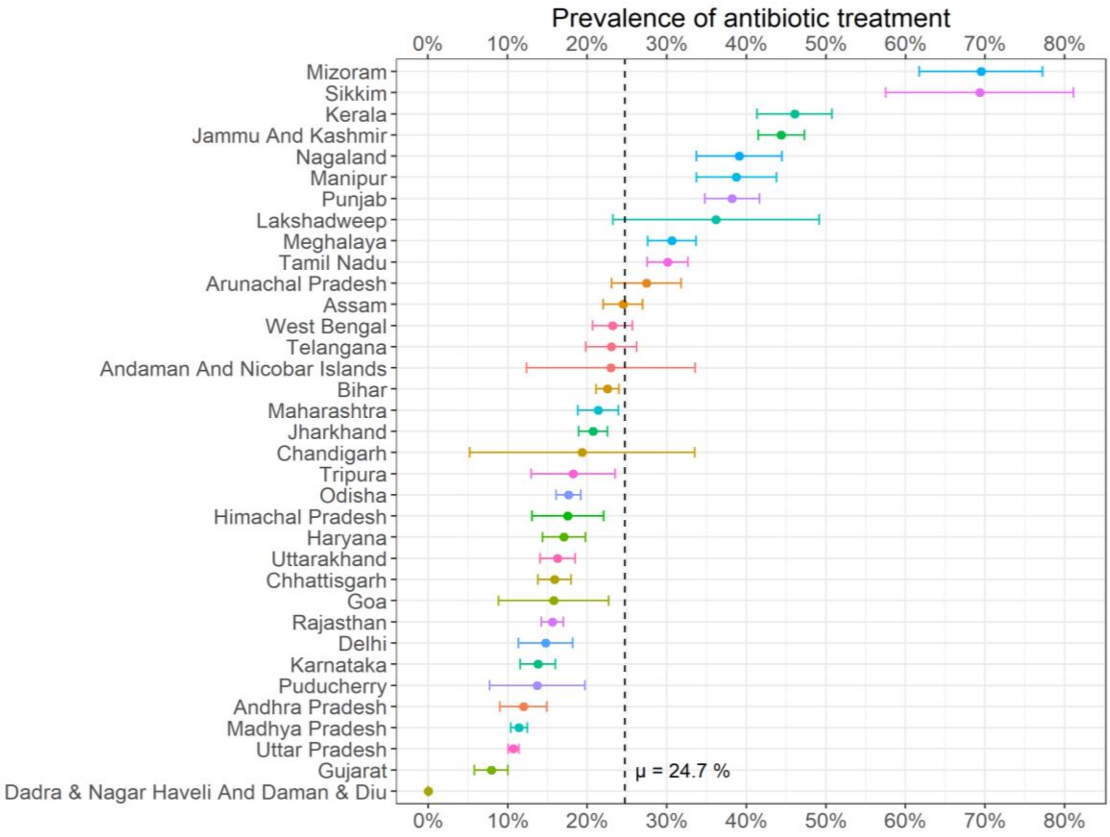

Figure S3. Prevalence of under-three-year-old children who received antibiotics to treat acute respiratory infection symptoms according to the state or union territory. Bars correspond to 95% confidence intervals.

Table S4. Prevalence of antibiotic treatment for under-three-year-old Indian children with symptoms of ARI in the previous two weeks by zones and state/union territory; NFHS-4 and NFHS-5; N = 17,452.

	Received antibiotics	
	**No**<br>N = 14,186 (81.3%)[1]	**Yes**<br>N = 3,266 (18.7%)[1]
**Zones**		
Southern	3,486.9 (75.6%)	1,126.0 (24.4%)
Western	2,954.5 (86.6%)	458.6 (13.4%)
Northern	1,215.6 (76.5%)	373.8 (23.5%)
Central	3,347.0 (85.8%)	554.0 (14.2%)
Eastern	1,886.6 (78.4%)	519.0 (21.6%)
Northern Eastern	1,295.7 (84.7%)	234.2 (15.3%)
**State/Union territory**		
Andaman and Nicobar Islands	1.9 (77.0%)	0.6 (23.0%)
Andhra Pradesh	356.7 (88.0%)	48.5 (12.0%)
Arunachal Pradesh	5.5 (72.6%)	2.1 (27.4%)
Assam	232.6 (75.5%)	75.4 (24.5%)
Bihar	2,378.7 (77.5%)	691.4 (22.5%)
Chandigarh	6.5 (80.6%)	1.6 (19.4%)
Chhattisgarh	261.8 (84.1%)	49.4 (15.9%)
Dadra & Nagar Haveli and Daman & Diu	2.4 (100.0%)	0.0 (0.0%)
Goa	13.2 (84.2%)	2.5 (15.8%)
Gujarat	339.7 (92.1%)	29.2 (7.9%)
Haryana	232.8 (82.9%)	47.9 (17.1%)
Himachal Pradesh	35.4 (82.4%)	7.5 (17.6%)
Jammu And Kashmir	73.8 (55.6%)	58.9 (44.4%)
Jharkhand	447.1 (79.3%)	117.0 (20.7%)
Karnataka	273.0 (86.2%)	43.6 (13.8%)
Kerala	120.3 (53.9%)	102.7 (46.1%)
Lakshadweep	0.5 (63.8%)	0.3 (36.2%)
Madhya Pradesh	910.0 (88.6%)	117.4 (11.4%)
Maharashtra	1,072.3 (78.6%)	291.7 (21.4%)
Manipur	15.4 (61.3%)	9.7 (38.7%)
Meghalaya	44.2 (69.4%)	19.5 (30.6%)
Mizoram	1.3 (30.5%)	3.0 (69.5%)
Nagaland	4.8 (60.9%)	3.1 (39.1%)
Delhi	219.2 (85.2%)	38.0 (14.8%)
Odisha	670.5 (82.4%)	143.5 (17.6%)
Puducherry	18.6 (86.3%)	2.9 (13.7%)
Punjab	178.3 (61.8%)	110.2 (38.2%)
Rajasthan	819.1 (84.4%)	151.3 (15.6%)
Sikkim	0.2 (30.7%)	0.5 (69.3%)
Tamil Nadu	659.0 (69.9%)	283.7 (30.1%)
Tripura	24.2 (81.8%)	5.4 (18.2%)
Uttar Pradesh	3,378.4 (89.3%)	406.1 (10.7%)
Uttarakhand	159.4 (83.7%)	31.0 (16.3%)
West Bengal	814.5 (76.8%)	246.0 (23.2%)
Telangana	415.1 (77.0%)	124.2 (23.0%)

[1]Frequency (%)

ARI: acute respiratory infection
